# Out-of-plane chiral domain wall spin-structures in ultrathin in-plane magnets

**DOI:** 10.1038/ncomms15302

**Published:** 2017-05-19

**Authors:** Gong Chen, Sang Pyo Kang, Colin Ophus, Alpha T. N'Diaye, Hee Young Kwon, Ryan T. Qiu, Changyeon Won, Kai Liu, Yizheng Wu, Andreas K. Schmid

**Affiliations:** 1 NCEM, Molecular Foundry, Lawrence Berkeley National Laboratory, Berkeley, California 94720, USA; 2 Department of Physics, Kyung Hee University, Seoul 02447, Korea; 3 Advanced Light Source, Lawrence Berkeley National Laboratory, Berkeley, California 94720, USA; 4 Physics Department, University of California, Davis, California 95616, USA; 5 Department of Physics, State Key Laboratory of Surface Physics and Collaborative Innovation Center of Advanced Microstructures, Fudan University, Shanghai 200433, China

## Abstract

Chiral spin textures in ultrathin films, such as skyrmions or chiral domain walls, are believed to offer large performance advantages in the development of novel spintronics technologies. While in-plane magnetized films have been studied extensively as media for current- and field-driven domain wall dynamics with applications in memory or logic devices, the stabilization of chiral spin textures in in-plane magnetized films has remained rare. Here we report a phase of spin structures in an in-plane magnetized ultrathin film system where out-of-plane spin orientations within domain walls are stable. Moreover, while domain walls in in-plane films are generally expected to be non-chiral, we show that right-handed spin rotations are strongly favoured in this system, due to the presence of the interfacial Dzyaloshinskii–Moriya interaction. These results constitute a platform to explore unconventional spin dynamics and topological phenomena that may enable high-performance in-plane spin-orbitronics devices.

## Abstract (web summary)

Chiral domain walls in magnetic films can be electrically controlled, which makes them attractive for applications, but domain walls in ultrathin films are normally non-chiral. Here, the authors observe chiral domain walls in ultrathin Fe/Ni bilayers that are stabilized by the magnetic anisotropy.

Spin textures in magnetic materials are determined by the balance of contributions to the magnetic free energy. In-plane magnetized films are a textbook case^1^: it is generally expected that ultrathin films have Néel wall textures where the spin vector rotates within the plane^2^, and thicker films have Bloch wall textures where spins tilt through out-of-plane alignments^3^,^4^. Such film thickness-dependent domain wall-type transitions have been studied in various systems^1^,^5^,^6^,^7^, for example the transition is at ∼30–35 nm thickness in permalloy films^8^. The domain wall transitions are a result of stray field and anisotropy energies competing along two possible hard axes^1^,^9^.

Recently, homochiral spin textures have been observed in the presence of the interfacial Dzyaloshinskii–Moriya interaction (DMI)^10^,^11^,^12^,^13^,^14^,^15^,^16^,^17^. Such chiral spin textures have been shown to offer enormous performance advantages in electrically controlled magnetic devices, including extremely fast current-driven domain wall motion and low threshold current^18^. However, for in-plane magnetized ultrathin films the most common wall type, Néel walls, are expected to be non-chiral^1^. This is because effects of the DMI on in-plane Néel walls are considered to be unimportant. This assessment comes from a simple symmetry argument. The interfacial DMI term is written as 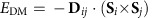, where **S**_*i*_ and **S**_*j*_ are two atomic spins on neighbouring atomic sites *i* and *j*, and **D**_*ij*_ is the DMI vector. For in-plane Néel walls the cross-product **S**_*i*_ × **S**_*j*_ points out-of-plane, and **D**_*ij*_ is a vector that lies within the plane^13^; therefore the contribution of the DMI to the free energy of the wall vanishes.

In atomically thin films, the interplay between strong interfacial DMI and exchange interaction can lead to homogeneous^14^,^15^ or inhomogeneous cycloidal spin spirals^16^. Driven by fundamental curiosity and the possibility of high-performance applications, it is interesting to explore other possibilities to stabilize chiral domain walls in in-plane magnetized thin films.

In the following, we show how the thickness-dependent Néel-to-Bloch wall transition can be reversed by deliberately adjusting magnetic anisotropies, that is, stabilizing out-of-plane domain walls in ultrathin films. Moreover, we show that out-of-plane domain walls stabilized in this way are indeed right-handed chiral walls. Modelling the domain walls in Monte-Carlo simulations, we show that their stability can be understood by taking into account a uniaxial anisotropy contribution, a perpendicular anisotropy contribution as well as the contribution of the interfacial DMI.

## Results

### Observation of the inverse Néel–Bloch transition

**Figure 1:**
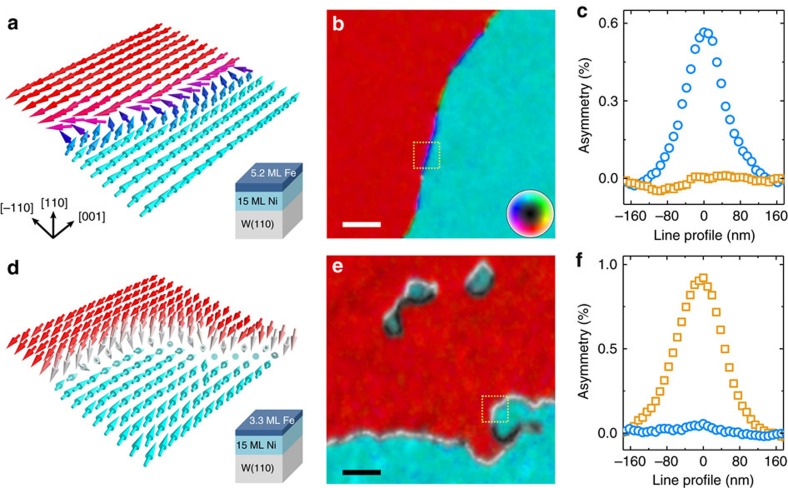
Real-space observation of novel domain wall structures. (**a**) Pixel-by-pixel magnetization vector map shows typical in-plane Néel texture of domain wall in a 350 × 350 nm region of 5.2 ML Fe/15 ML Ni/W(110) bilayer (sample structure sketched in inset). (**b**) Compound SPLEEM image shows larger area (dashed yellow square outlines the region shown in **a**); in-plane orientation of the magnetization is rendered as colour according to colour wheel in the inset, with +*z*/−*z* out-of-plane component rendered as brightness (black/white). Scale bar, 500 nm. (**c**) Averaged line profile of out-of-plane (orange squares) and in-plane (blue circles) magnetization components across domain wall in **b**. (**d**) Pixel-by-pixel magnetization vector map, showing unexpected out-of-plane domain wall in a 350 × 350 nm region of 3.3 ML Fe/15 ML Ni/W(110) bilayer (sample structure sketched in inset). (**e**) Compound SPLEEM image shows larger area (dashed yellow square outlines the region shown in **d**). Scale bar, 500 nm. (**f**) Averaged line profile of out-of-plane (orange squares) and in-plane (blue circles) magnetization components across domain wall in **e**.

Spin-polarized low-energy electron microscopy (SPLEEM)^19^,^20^ was used to measure maps of the orientation of the magnetization vector in ultrathin, single-crystalline Fe/Ni bilayers grown on a W(110) crystal. The characteristic spin texture of a domain wall in a bilayer composed of 15 atomic monolayers (ML) of nickel and 5.2 ML of iron is shown in Fig. 1a,b. In this sample the two-fold easy magnetization axis is along [001]; low-index directions are indicated by black arrows. Rendering SPLEEM measurements as an arrow-array, Fig. 1a represents the magnetization in a 375 nm × 375 nm region. Here a 180° domain wall separates a domain on the left that is magnetized in the [00–1] direction from a domain on the right that is magnetized in the [001] direction. Magnetization inside the domain wall rotates within the film plane, as expected in a conventional Néel wall^1^,^2^. SPLEEM data of a larger area surrounding this region (dashed yellow box) are rendered as a colour plot in Fig. 1b, where the magnetization direction measured in each pixel is represented as colour according to the colour wheel in the inset (red—[00–1], cyan—[001], green/yellow—[1–10], purple—[–110]). Colour variation along the domain wall (green/yellow versus purple sections) indicates that the sense of spin rotation within the wall randomly reverses in various places. Figure 1c plots independent SPLEEM measurements of the in-plane and out-of-plane magnetization components along a trajectory crossing the domain wall, corroborating the in-plane texture of this domain wall (see Methods for details). Taken together, the data reproduced in Fig. 1a–c shows that, unsurprisingly, the domain wall has a Néel wall structure throughout and there is no chiral preference in the magnetic structure of this film^1^.

A much more unusual result emerges when the thickness of the Fe layer is slightly less, 3.3 ML as sketched in Fig. 1d. In this case, SPLEEM data rendered as arrow-array in Fig. 1d highlights that the domain wall has a spin texture that is in stark contrast with the conventional Néel wall described above: within the 180° domain wall separating a domain on the left/back (magnetized in the [00–1] direction) from a domain on the right/front (magnetized in the [001] direction) the spins rotate through an out-of-plane alignment. Using the same colour wheel as before, Fig. 1e renders SPLEEM data of a larger area surrounding this region (355 nm × 355 nm, dashed yellow box) as a colour plot. In this case pixels within the domain wall appear as white or black, indicating that the magnetization direction within domain walls always rotates towards out-of-plane directions. Note that in some pixels in Fig. 1d,e the magnetization tilts slightly towards out-of-plane, which may result from additional perpendicular anisotropy induced by substrate roughness (see LEEM morphology in Supplementary Fig. 1d). This additional small anisotropy may influence spin textures when the system is close to a spin reorientation transition. Figure 1f plots independently the in-plane and out-of-plane magnetization components along a trajectory crossing the domain wall, and further supports the out-of-plane texture of this domain wall. Taking into account the fact that the width of this domain wall (full width at half maximum ∼110 nm in Fig. 1f) is much larger than the thickness of the film (a few nm), the domain wall texture observed in Fig. 1d–f should be energetically unfavourable due to the stray field energy^1^,^2^.

### Possible origin of the observed domain wall

Now we discuss how the stability of this unusual domain wall spin texture can be understood. In the Fe/Ni/W(110) system, additional contributions to the magnetic free energy might play a role to stabilize the domain wall structure. These include perpendicular magnetic anisotropy of the Fe layer^21^,^22^, uniaxial in-plane anisotropy due to epitaxial strain at the Ni(111)/W(110) interface^21^,^22^ and the interfacial DMI at the Ni/W interface^22^. Looking at Fig. 1a,b provides one hunch. The in-plane uniaxial anisotropy *K*_u_, stabilizing the magnetization direction of the two domains, is along the W[001] direction; so the spins within this conventional Néel wall point into a hard-axis direction along W[1–10]. However, in most thin film systems the energy penalty associated with rotating spins into a hard axis is smaller than the dipolar energy penalty associated with rotating spins into an out-of-plane alignment. One contribution that can counter this dipolar energy penalty is perpendicular magnetic anisotropy, which is indeed present in Fe layers grown in this orientation^21^,^22^.

The strength of this perpendicular anisotropy can be demonstrated by measuring the magnetization direction in Fe/Ni/W(110) as a function of the Fe overlayer thickness (Supplementary Fig. 2). In the absence of a Fe overlayer, 15 ML Ni/W(110) films are in-plane magnetized. The magnetization starts to cant towards the out-of-plane direction at 0.5 ML Fe coverage. This canting gradually increases and reaches fully out-of-plane magnetization around *d*_Fe_=2.4 ML. With additional Fe, the magnetization gradually cants back towards in-plane orientation, until the double-spin reorientation transition is complete at *d*_Fe_=3.1 ML. In this case of 15 ML Ni/W(110), the range of Fe film thickness *d*_Fe_ resulting in stable out-of-plane domain walls is estimated to be between 3.1 ML and 4 ML Fe (Supplementary Fig. 3). Similar out-of-plane wall textures are also observed with other Ni layer thickness in the range between 3 ML and 20 ML (with corresponding adjustments of the Fe layer thickness).

In this picture of the Fe/Ni/W(110) system, the dipolar penalty for out-of-plane spin alignment is largely balanced by the perpendicular anisotropy of the Fe layer and, as a result, out-of-plane domain wall spin texture may be energetically favoured over conventional Néel wall texture.

### Simulations for the out-of-plane domain wall

**Figure 2:**
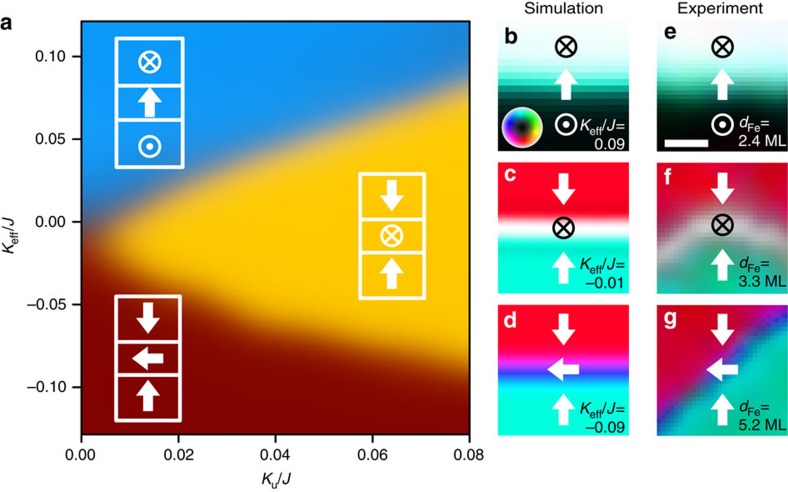
Phase diagram of domain wall types. (**a**) Phase diagram of domain walls in *K*_eff_−*K*_u_ space. *K*_eff_ corresponds to effective anisotropy, and *K*_u_ corresponds to uniaxial anisotropy. In the region shaded in brown the ground state is in-plane Néel wall. Region in gold corresponds to out-of-plane walls separating in-plane domains and in the region shaded in blue Néel walls separate out-of-plane magnetized domains. Boundaries between three phases indicate the gradual transitions of domain wall type. (**b**–**d**) Simulated domain wall types match experimental observation, *K*_u_/*J*=0.05 (To enhance visibility of domain wall spin textures, 20 by 20 for **b** and 60 by 60 spin block arrays for **c**,**d** were cropped from simulated 200 by 200 arrays). (**e**–**g**) SPLEEM images of domain walls by in Fe/Ni bilayer on W(110), *d*_Fe_=2.4 ML, 3.3 ML, 5.2 ML respectively. Symbols indicate magnetization directions. (**e**–**g**) Scale bar (in **e**) 100 nm.

To test this hypothesis, we used Monte-Carlo simulations to compute ground-state domain wall spin textures as a function of the uniaxial anisotropy *K*_u_ and the total effective anisotropy *K*_eff_. Results, summarized in Fig. 2a, show that three phases can be distinguished, where domain wall ground states have different spin textures. When *K*_u_ is negligible then, as a function of increasing *K*_eff_, there is a spin reorientation transition where the in-plane easy magnetization axis (shaded brown in Fig. 2a), with domain walls exhibiting Néel wall texture, reorients to an out-of-plane easy axis (shaded blue in Fig. 2a), also with Néel type domain walls^17^,^22^,^23^,^24^. This is consistent with experimental observations of domain wall textures near spin reorientation transitions in Fe/Ni bilayers on Cu(100) crystals where *K*_u_≈0 (ref. 17) (Supplementary Fig. 4). When *K*_u_ is larger, a new phase appears (shaded gold in Fig. 2a) where the easy magnetization axis is in-plane, while spins within the domain walls rotate through out-of-plane alignments. Note that the strength of the DMI is considered in the simulation (Methods), and its value only slightly affects the boundary position (Supplementary Fig. 5).

Detailed domain wall spin textures predicted from Monte-Carlo simulations as a function of *K*_eff_ (Fig. 2b–d) are in excellent agreement with detailed experimental images of domain wall spin textures where *K*_eff_ was tuned by changing the thickness *d*_Fe_ of the Fe layer (Fig. 2e–g).

### Chirality of the out-of-plane domain walls

Inspection of a large quantity of SPLEEM images of Fe/Ni/W(110) films similar to the sample of Fig. 1d–f reveals an additional interesting feature of the out-of-plane domain walls. Surprisingly, the handedness of these rotating spin textures is not random. This is unusual because in conventional Néel walls, such as those shown in Fig. 1a–c, the handedness of spin rotations within the walls is expected (and observed) to be energetically degenerate: for example, in Fig. 1b left-handed and right-handed sections of domain wall (purple and green/yellow) alternate at random. In contrast, handedness of the out-of-plane domain walls we observe in thinner Fe/Ni/W(110) structures follows a systematic pattern (Fig. 1e). Propagating along the W[001] direction from domains magnetized along [001] (cyan in the figures) to domains magnetized along [00–1] (red in the figures), the domain wall magnetization always points down (white in the figures); and propagating from domains magnetized along [00–1] to domains magnetized along [001] the magnetization always points up (black in the figures). This pattern in the handedness of the spin textures of these domain walls suggests that the interfacial DMI at the Ni/W interface^22^ plays an important role in these structures.

Previously, effects of the interfacial DMI on in-plane magnetized systems have not been considered to be important^1^, because conventional in-plane Néel walls are always non-chiral (as discussed in Introduction). In contrast, for the in-plane magnetized system observed here the role of the interfacial DMI cannot be neglected because, within these domain walls, the cross-product **S**_*i*_ × **S**_*j*_ has finite components within the plane, leading to a finite magnitude of *E*_DM_.

**Figure 3:**
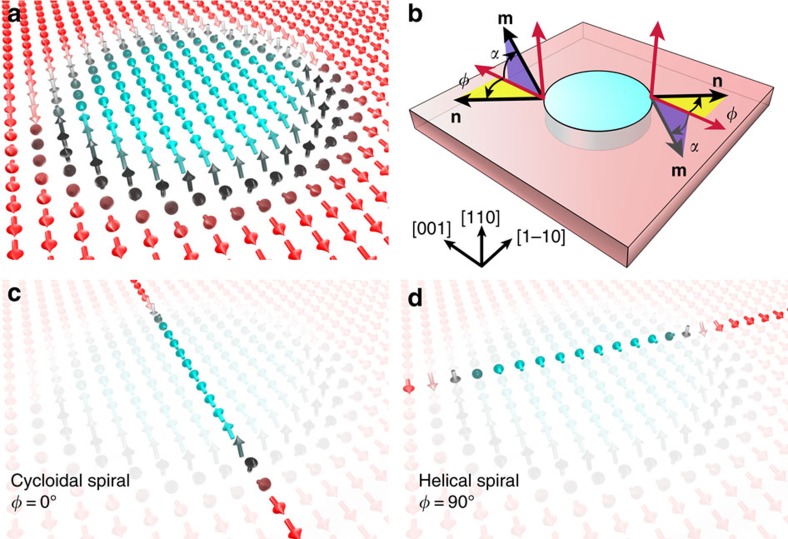
Sketch of different spin spiral types along different in-plane directions. (**a**) Sketch of spin blocks similar to experimental observation, compare cyan bubble domain in Fig. 1e. Each arrow indicates the direction of local magnetization. (**b**) Definitions of angles: *α* is angle between W[001] and domain wall magnetization **m** (see regions in purple), *φ* is angle between W[001] and domain wall normal vector **n** (see regions in yellow). Two sets of angle definitions are given on each side of the cyan domain, showing the meaning of the positive angle *α* and angle *φ.* (As sketched on opposite sides of the bubble domain, the vectors **n** and **m** reverse direction). (**c**) A cycloidal type spin spiral is highlighted for *φ*=0°. (**d**) A helical type spin spiral is highlighted for *φ*=90°.

The spin structure in the domain wall surrounding a circular bubble domain is a convenient shorthand to describe the chiral magnetic properties of this system. Figure 3a sketches the spin texture of a circle-shaped domain, essentially modelling the domain at the top, right of centre, in Fig. 1e. Trajectories across such a bubble domain along two orthogonal in-plane directions can be visualized as two types of spin spirals, that is, cycloidal (Néel-type) spin spirals along W[001] (Fig. 3c) and helical (Bloch-type) spin spirals along W[1–10] (Fig. 3d). Within this picture we can quantify the magnetic chirality of the system. To start, we define the angle *α* between the W[001] direction and the magnetization direction at points within domain wall, and the angle *φ* between the W[001] direction and the domain wall normal vector **n**. Cycloidal spin spirals (along W[001]) and helical spin spirals (along W[1–10]) are then associated with the values *φ*=0 and *φ*=90**°**, respectively, as sketched in Fig. 3c,d.

**Figure 4:**
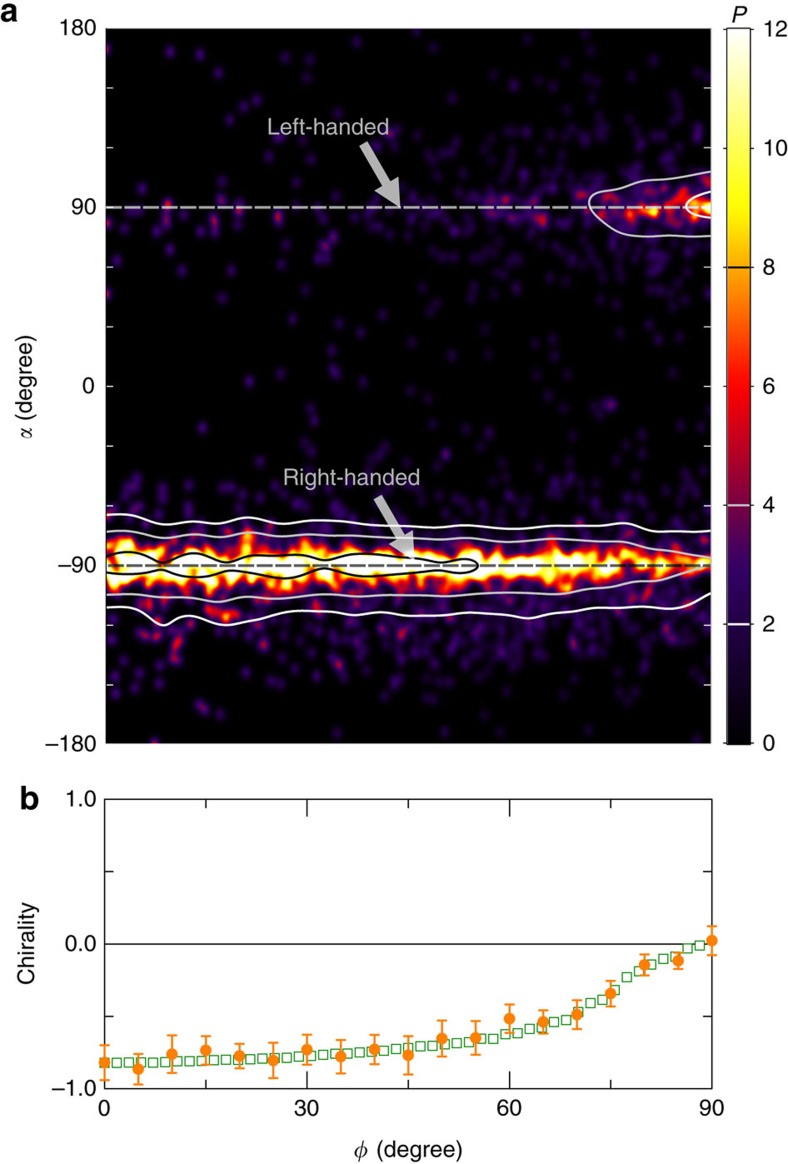
Domain wall chirality as a function of domain wall orientation. (**a**) Two-dimensional histogram of domain wall magnetization direction in *α*–*φ* space (see main text and Fig. 3b for definition of *α* and *φ*). Colour scale corresponds to prevalence of observed domain wall magnetization as a function of *α* and *φ*; scale *P* is normalized to units of multiples of random distribution along the vertical direction. White, grey and black solid lines correspond to *P*=2, *P*=4 and *P*=8, respectively. *α*=+90° and −90° highlighted by dashed lines indicate left-handed chirality and right-handed chirality, respectively. (**b**) *φ*-dependent chirality: experimental measurement of wall handedness from histogram **a** (orange solid dots), and domain wall handedness computed from Monte-Carlo simulations (green squares). The error bars are the s.d. of the fitting in Supplementary Fig. 6. *y*=+1 indicates left-handedness, *y*=−1 indicates right-handedness.

Next, the angles *α* and *φ* were measured in a large ensemble of domain walls. The results are summarized in the histogram plotted in Fig. 4a, showing the statistical probability of domain magnetization directions in *α–φ* space. Brighter/darker colour indicates higher/lower count for *α*/*φ* combinations. A horizontal bright streak at *α*=−90° spans the entire range of 0°<*φ*<90°, indicating the prevalence of right-handed domain wall spin textures with significant cycloidal character. Left-handed textures (*α*=90°) are more rare and are essentially limited to a narrow band near *φ*=90°, where the spin spiral has significant helical character. For *φ*=90°, where the spin spiral is pure helical type, *α*=+90° and −90° have comparable probability: this indicates that these domain walls are non-chiral. This difference of the chirality along different substrate directions is related to the orientation of the DMI vector **D**_*ij*_. Interfacial DMI vectors are generally oriented perpendicular to the atomic position vectors **r**_*ij*_=**r**_*i*_−**r**_*j*_ (refs 10, 11, 12, 13). The cross-product **S**_*i*_ × **S**_*j*_ is perpendicular/parallel to **r**_*ij*_ for cycloidal/helical type, as a result the DMI energy vanishes for helical textures^22^. The *φ*-dependent magnetic chirality apparent in Fig. 4a, featuring a gradual transition from chiral to non-chiral (Fig. 4b) as a function of increasing helical and decreasing cycloidal character of the domain walls (Supplementary Fig. 6), can be accurately reproduced by Monte-Carlo simulations (Methods). We note that without significant DMI, the *φ-*dependent cycloidal/helical transition remains but chirality disappears (Supplementary Fig. 7). This condition may be achieved experimentally by growing multilayers with inversion symmetric stacking sequences or by tailoring the DMI at interfaces by combining different heavy metals with opposite sign of the DMI.

### Stability of chiral out-of-plane walls in nanowires

**Figure 5:**
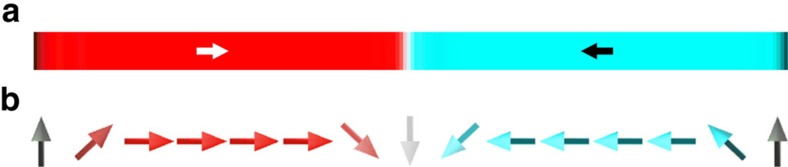
Simulated chiral domain wall in in-plane magnetized nanowire. Shape-induced uniaxial anisotropy provides hard axis in the direction across the nanowire, supporting the stability of chiral out-of-plane domain walls even in the absence of uniaxial magnetocrystalline anisotropy. (**a**) Top view of the simulated spin configuration in the nanowire. Arrows indicate the orientation of the magnetization in the domain. (**b**) Side view of the nanowire.

The Slonczewski-like torque that drives fast chiral domain wall motion in perpendicularly magnetized spin-orbitronics systems^23^,^25^,^26^,^27^ vanishes for conventional in-plane Néel wall textures (such as transverse walls in nanowire geometries)^25^. But out-of-plane domain wall spin textures with DMI-stabilized chirality, as seen here in the Fe/Ni/W(110) system, may offer important benefits for current-driven domain wall dynamics. Furthermore, one key ingredient in stabilizing these out-of-plane domain wall textures—a uniaxial anisotropy *K*_u_—is present in nanowires even in absence of the uniaxial anisotropy provided by the W(110) substrate: considering that the shape-induced uniaxial anisotropy provides a hard axis in the direction across the nanowire^28^, it is interesting to consider whether a tailored addition of perpendicular anisotropy to in-plane magnetized nanowires could result in domain wall textures similar to those we observed in the Fe/Ni/W(110) films. To provide an estimate of this possibility, we modelled such a nanowire in a Monte-Carlo simulation. A simulated spin structure in a nanowire with 200 × 10 spin blocks is shown in Fig. 5. This result shows that, with dimensionless values (expressed as fractions of the exchange interaction *J*) for the effective anisotropy *K*_eff_/*J*=−0.01, nanowire shape-induced uniaxial anisotropy *K*_u_/*J*=0.476, and strength of the DMI **D**_*ij*_/*J*=0.1, we find that right-handed chiral out-of-plane domain walls have the lowest energy in this nanowire. In the presence of the giant spin Hall effect of tungsten^29^ and strength of the DMI similar as in a number of systems (the strength of the DMI found here, ∼0.53 meV per atom (Methods), is of the same order as in other reported systems^17^,^18^,^22^,^24^,^26^,^27^, the Slonczewski-like torque exerts a significant force that may enable fast current-driven domain wall propagation, extending the possibility of efficient electrical control of magnetic structures^23^,^25^,^26^,^27^,^30^ to in-plane magnetized systems.

## Conclusions

In summary, using micromagnetic imaging experiments we found an unusual type of chiral out-of-plane domain wall spin textures in an in-plane magnetized ultrathin film system. Supported by Monte-Carlo simulation, the interplay between magnetic anisotropies is understood to be the driving force stabilizing the out-of-plane wall textures. The magnetic chirality of these domain walls is stabilized along in-plane orientations and is understood to result from the interfacial DMI. Current-31,32,33,34,35,36,37,38 and field-driven^39^,^40^,^41^,^42^,^43^,^44^ domain wall dynamics in in-plane magnetized nanowires has been an important research area. The out-of-plane domain wall structures described in this paper may provide a platform to explore unconventional spin dynamics and topological phenomena^45^,^46^,^47^,^48^ that may enable high-performance in-plane magnetized memory^49^ and logic devices^50^,^51^.

## Methods

### Sample preparation

The W(110) substrate was cleaned by flash heating to 1,950 °C in 3 × 10^−8^ Torr O_2_, and final annealing at the same temperature under ultrahigh vacuum. Fe and Ni layers were deposited at room temperature by physical vapour deposition from electron beam evaporators, and the Fe film thickness *d*_Fe_ and Ni film thickness *d*_Ni_ was controlled by monitoring the image intensity oscillations associated with atomic layer-by-layer growth. The sample was annealed to 630 °C for several minutes after growth of the first 1 ML Ni to develop the well-ordered c-(1 × 7) structure as a template for subsequent growth of Ni film and Fe film in fcc(111) epitaxy^52^.

### Magnetic imaging and vector field analysis

Magnetic images were measured using the SPLEEM instrument at the National Center for Electron Microscopy at Lawrence Berkeley National Laboratory^17^,^20^,^22^,^24^. The contrast in SPLEEM images represents the asymmetry of the spin-dependent reflection between spin-polarized beams with opposite polarization (up and down), which can be written as 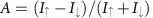. For a selected axis of the illumination spin direction, this asymmetry *A* is proportional to the strength of the component of the magnetization **M** along the selected axis. The Cartesian components *M*_*x*_, *M*_*y*_ and *M*_*z*_ of the magnetization were resolved by recording triplets of images with the electron beam spin polarization aligned along the *x*, *y* and *z* directions, respectively^53^. All the SPLEEM images were measured on the as-grown samples at room temperature. The energy of the incident electron beam was chosen to be 7.5 eV to maximize the magnetic contrast. Supplementary Fig. 1a–c shows typical images. High signal-to-noise ratio was obtained by averaging sets of 150 images for each Cartesian direction, after application of a drift correction routine based on tracking the positions of the atomic steps in the images. The magnetization vector is represented in hue satutation lightness (HSL) colour space where the in-plane magnetization direction is mapped on hue and the out-of-plane angle is mapped on brightness (Fig. 1b,e, Supplementary Fig. 1e). Figure 2e–g is cropped from 10° rotated Supplementary Fig. 8a, 1e and 8c, respectively. The *α*–*φ* histogram in Fig. 4a is plotted in a same way as described in ref. 22. To achieve better statistic, the *φ* angle was separated into bins of 5°. In each bin, the average value of the magnetic chirality was estimated using the method shown in Supplementary Fig. 6.

### Monte-Carlo simulation

The Monte-Carlo simulations were carried out based on a two-dimensional model described in refs 54, 55, where exchange interaction, dipolar interaction, magnetic anisotropy, in-plane uniaxial magnetic anisotropy as well as the DMI are considered^22^.

The total energy term can be written as


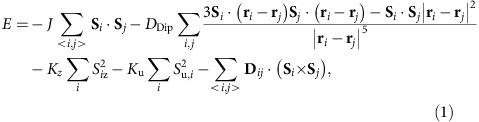


where **S**_*i*_ and **S**_*j*_ are spin moments located on atomic sites *i* and *j* in a two-dimensional plane, **r**_*i*_ and **r**_*j*_ are the position vectors of the spin blocks in sites *i* and *j*. *J*, *D*_Dip_, *K*_*z*_, *K*_u_ and **D**_*ij*_ correspond to exchange interaction, dipole interaction, perpendicular anisotropy, uniaxial in-plane anisotropy and DMI, respectively. The effective anisotropy *K*_eff_ is defined as 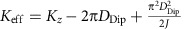. The dimensionless parameters *J*, *D*_Dip_, *K*_*z*_, *K*_u_ and **D**_*ij*_ are used for simulating domain wall spin structures. For the simulation results summarized in Fig. 2, the values *J*=1, *D*_Dip_/*J*=0.1, *D*_*ij*_/*J*=0.1 were assumed, and 
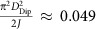
 (ref. 54). For the results summarized in Fig. 4b, the values *J*=1, *D*_Dip_/*J*=0.1, *K*_eff_/*J*=−0.01, *K*_u_/*J*=0.05 and *D*_*ij*_/*J*=0.1 were used. As described in ref. 17, system temperature is represented by allowing spins to fluctuate according to Boltzmann statistics. Phase diagram and domain configuration shown in Fig. 2a–d are simulated using 200 × 200 spin block arrays with periodic boundary condition applied for both directions^54^,^55^. To compute the energies of the three types of domain walls observed in our experiments (Néel wall separating out-of-plane domains, out-of-plane domain wall separating in-plane domains and in-plane Néel wall separating in-plane domains), spin block arrays are initialized to represent pairs of domains so that in each domain all spin blocks are aligned parallel to the easy magnetization direction (out-of-plane domains for Fig. 2b and head-to-head in-plane domains for Fig. 2c,d); and the two domains are separated by either in-plane or out-of-plane domain walls with a wall thickness of one spin block. The nanowire configuration shown in Fig. 5 is simulated using 200 × 10 spin block arrays with periodic boundary conditions only applied along nanowire direction (left and right side in Fig. 5a), where *D*_Dip_ is not considered, and *K*_u_/*J*=0.476 is deduced based on the demagnetization factor of the nanowire. Initial temperature in the simulation is chosen to be lower than the Curie temperature to avoid destroying the initial setting. After annealing these spin block arrays, domain walls relax to a finite thickness and the lowest energy state is determined by comparing the energies of the three states. *φ*-dependent chirality in Fig. 4b is simulated in arrays of 600 by 10 spin blocks separated by a horizontal domain wall^22^. Each trial proceeds from random starting configurations which are allowed to relax by gradually dropping the temperature from well above the Curie point to zero. Iterations are repeated until the total energy is stabilized for all simulations (128 trial number).

At W(110) interfaces, the DMI vector **D**_*ij*_ is perpendicular to the distance vector **R**_*ij*_=**R**_*i*_−**R**_*j*_ between sites *i* and *j* and, as a result of the bcc(110) interface, the DMI is anisotropic^13^ such that the DMI vector has a finite magnitude along W[–110] directions and vanishes along W[001] directions. The strength of *D*_Dip_ can be estimated as 
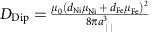
, where *μ*_0_=4π × 10^−7^ H m^−1^, *μ*_B_=9.274 × 10^−24^ A m^2^, *μ*_Ni_=0.6*μ*_B_, *d*_Ni_=15 ML, *μ*_Fe_=2.6*μ*_B_, *d*_Fe_=3.3 ML, *a*_||_=2.49 Å. We find 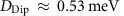 per atom. These simulations best match the observed dependence of domain wall handedness on domain wall orientation (Fig. 4b) when **D**_*ij*_=*D*_Dip_, which provides an experimental estimate of the strength of the DMI in this system of ∼0.53 meV per atom.

### Data availability

The data that support the findings of this study are available from the corresponding author upon request.

## Additional information

**How to cite this article:** Chen, G. *et al*. Out-of-plane chiral domain wall spin-structures in ultrathin in-plane magnets. *Nat. Commun.* **8,** 15302 doi: 10.1038/ncomms15302 (2017).

**Publisher's note:** Springer Nature remains neutral with regard to jurisdictional claims in published maps and institutional affiliations.

## Supplementary Material

Supplementary Information Supplementary Figures
