# Supplementary material for: Out-of-plane chiral domain wall spin-structures in ultrathin in-plane magnets
Source: Nat Commun. 2017 May 19;8:15302. doi: 10.1038/ncomms15302 (PMC5454456; doi:10.1038/ncomms15302)
Supplement: Supplementary Information — Supplementary Figures [file ncomms15302-s1.pdf]

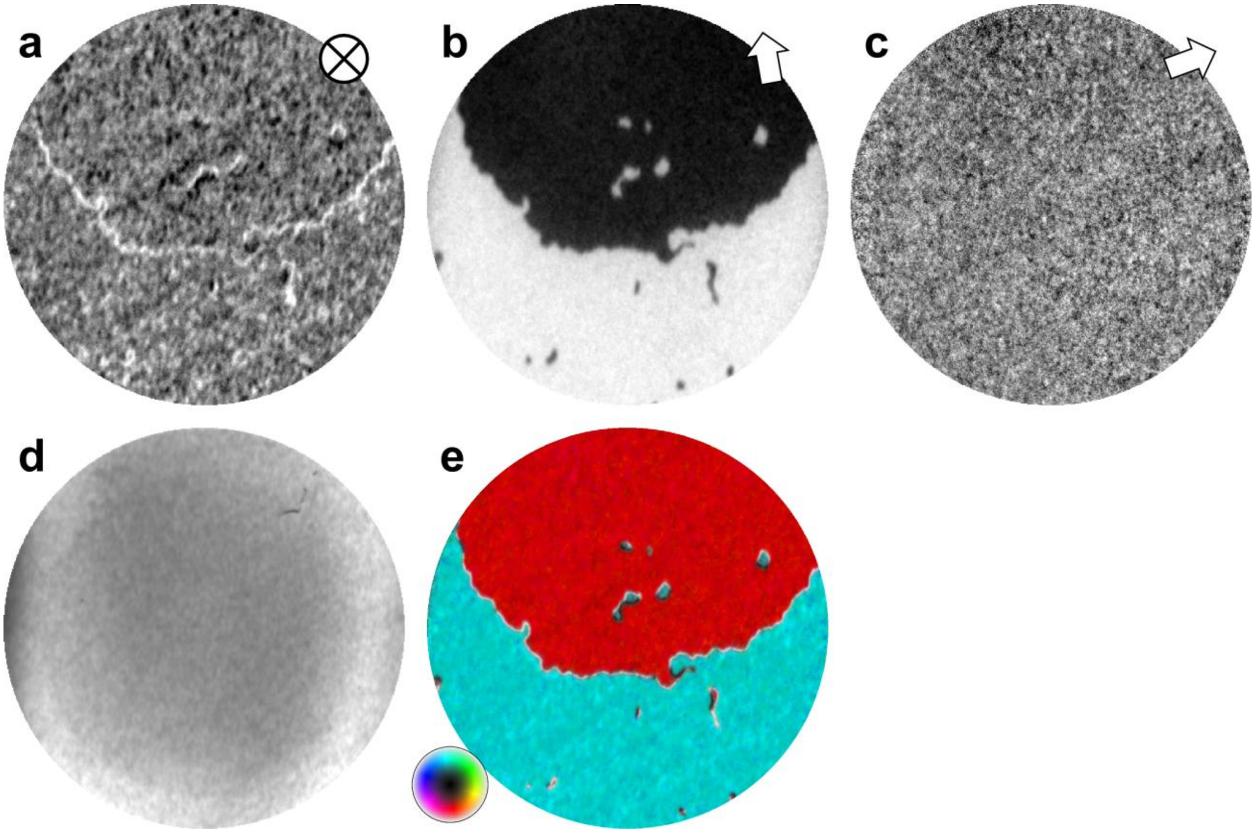


**Supplementary Figure 1 | Real-space observation of domain wall spin structure using SPLEEM.** (**a**)-(**c**) SPLEEM images of the 3.3ML Fe/15ML Ni/W(110) bilayers, mapping three orthogonal magnetization components. Symbols near upper right of **a**, **b**, **c** show spin polarization directions of the incident electron beam. (**d**) LEEM image on the same area. Field of view is 10 μm. (**e**) Compound SPLEEM image constructed from images **a**-**c**, highlighting the direction of spin vector within domain walls. Colour wheel shows in-plane orientation of the magnetization, and brightness shows +$z$/-$z$ out-of-plane component.

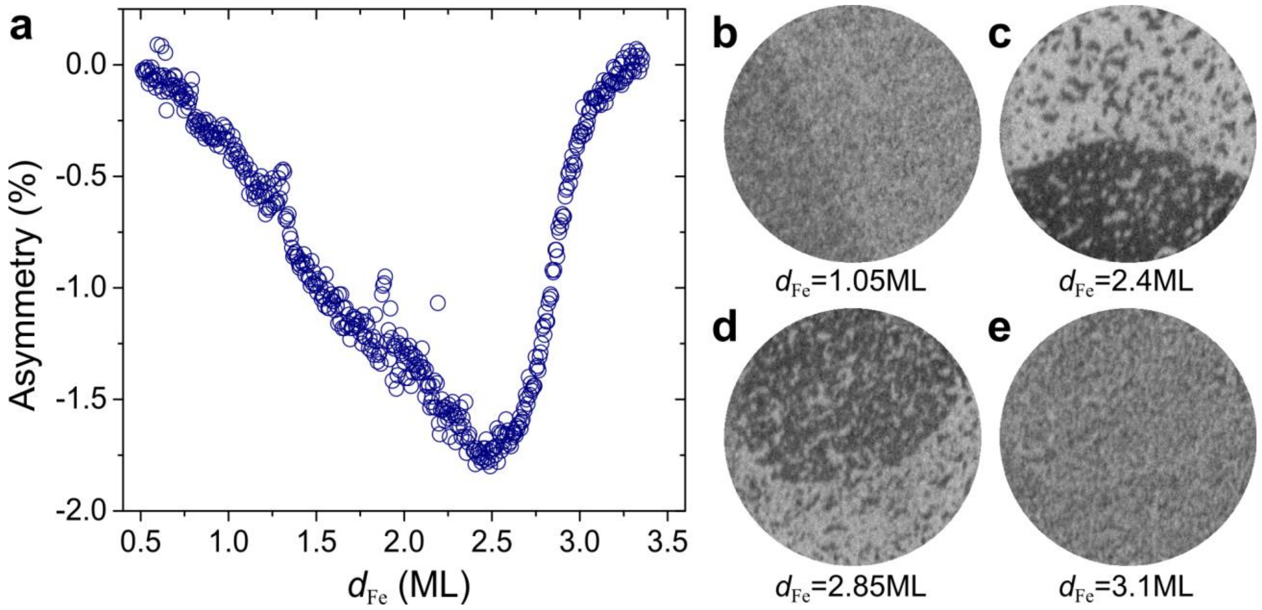


**Supplementary Figure 2 | Fe overlayer induced double spin reorientation transition on 15ML Ni/W(110).** (**a**) Spin-dependent reflectivity asymmetry (see Methods), averaged over black out-of-plane domains, as a function of Fe thickness $d_{Fe}$ (zero value of the asymmetry indicates in-plane orientation of the magnetization). (**b**)-(**e**) SPLEEM images indicate the evolution of out-of-plane magnetization component as a function of Fe thickness. These observations suggest that at $d_{Fe}$ = 3.3ML, after – but very close to – the spin reorientation transition, the strength (absolute value) of $K_{eff}$ is much smaller than well within the out-of-plane anisotropy region near $d_{Fe}$ = 2.4ML or well within the in-plane anisotropy region at $d_{Fe}$ = 5.2ML. Field of view of **b**-**e** is 10 $\mu$m.

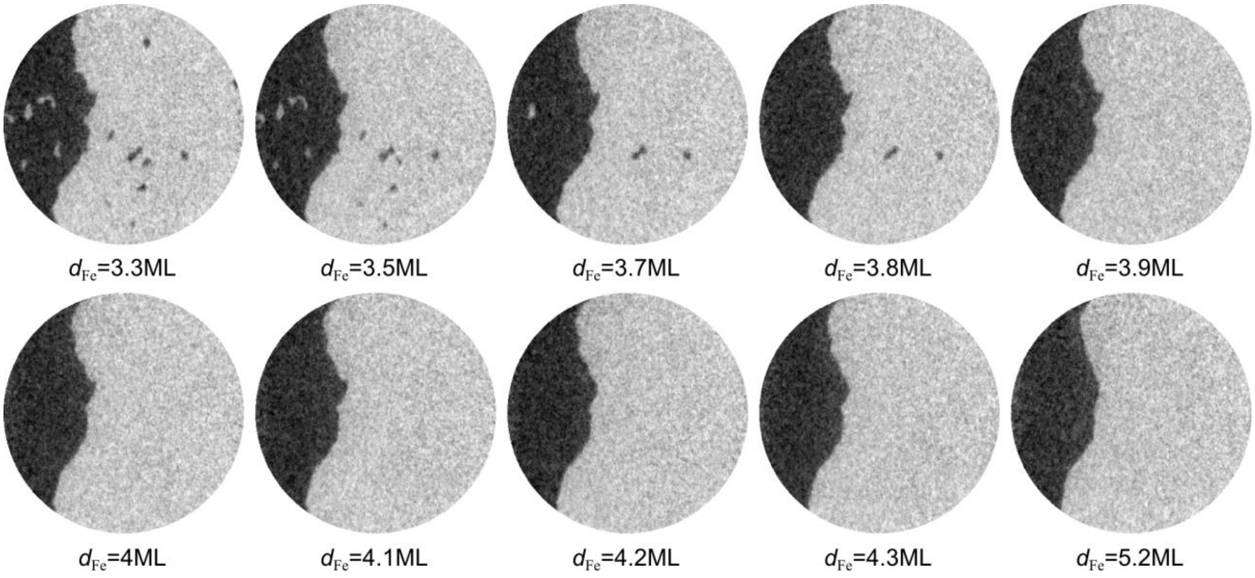


**Supplementary Figure 3 | Evolution of domain wall shape in Fe/15ML Ni/W(110) system as a function of Fe overlayer thickness $d_{Fe}$.** Domain wall type transition from Bloch- to Néel texture is accompanied with domain wall shape transition: sequence of SPLEEM images acquired with electron beam spin polarization parallel to W[001] (the easy axis of $K_u$) shows transition from rough (zigzag) domain wall shape and presence of small bubble domains to smooth domain wall shape and absence of bubble domains. Out-of-plane texture of domain walls in first image and in-plane Néel texture of domain wall in last frame is demonstrated in corresponding compound images reproduced in Supplementary Figure 8b and c, respectively. Significant straightening of domain wall shape in the range $d_{Fe}$=3.8ML to $d_{Fe}$=4.2ML suggests that the domain wall type transition from out-of-plane wall to in-plane Néel wall likely occurs near $d_{Fe}$=4ML. Field of view is 10 μm.

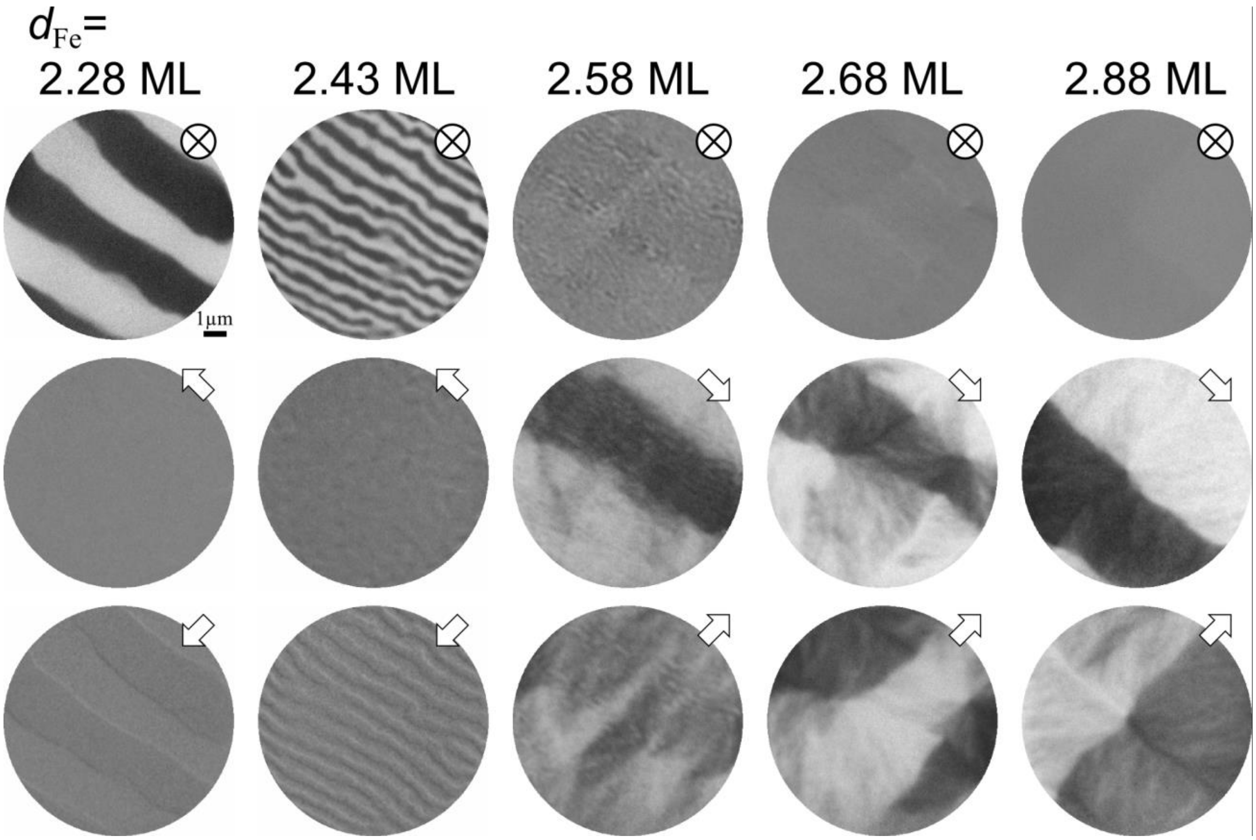


**Supplementary Figure 4 | Evolution of domain wall spin texture as a function of Fe layer thickness in the Fe/Ni/Cu(100) system.** SPLEEM images of the Fe/2ML Ni/Cu(001) system show that out-of-plane magnetic anisotropy is gradually reduced as a function of increasing Fe thickness $d_{Fe}$. Each column corresponds to a set of three SPLEEM images, recorded with three alignments of orientation of incident beam spin polarization as shown by arrows at the upper right in each image, under otherwise identical conditions. The Fe thickness $d_{Fe}$ is indicated in on top of each column. As the spin reorientation transition from out-of-plane to in-plane anisotropy occurs near $d_{Fe}$=2.58ML, the domain wall spin-texture transitions from chiral Néel walls in the out-of-plane stripe-domain phase (1$^{st}$ and 2$^{nd}$ column) to in-plane Néel walls (4$^{th}$ and 5$^{th}$ column). Field of view is 10 μm.

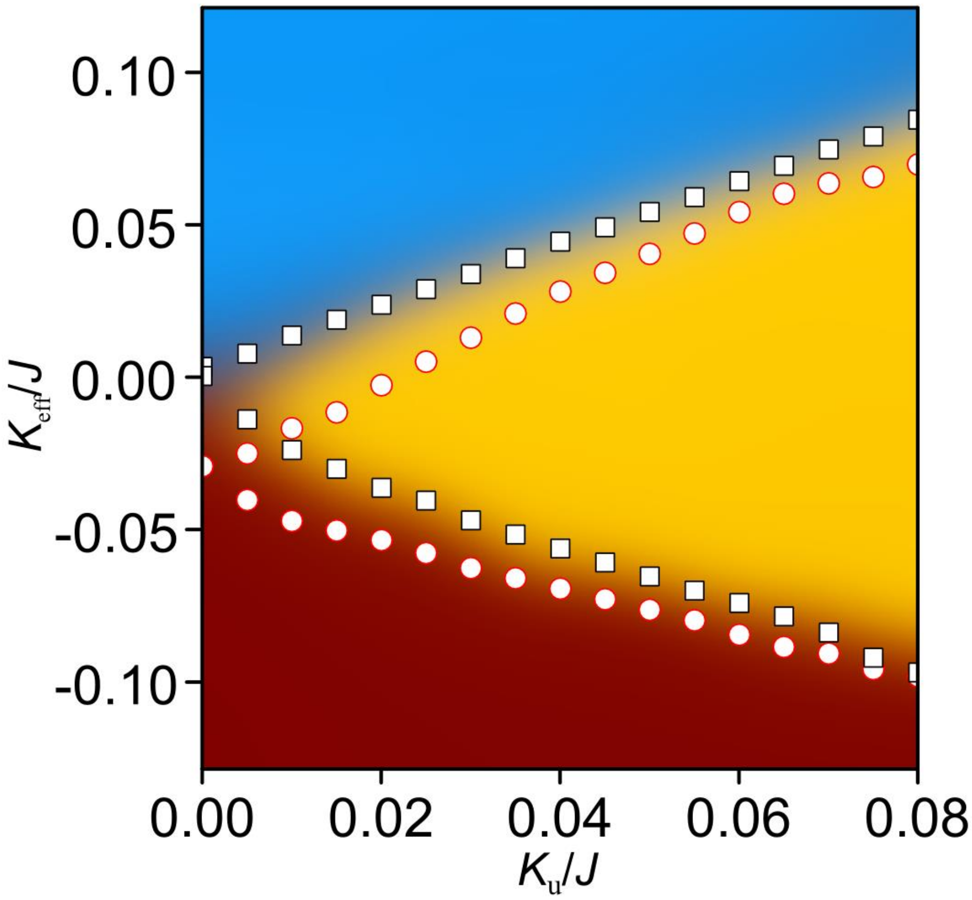


**Supplementary Figure 5 | Dependence of domain wall type phase diagram on the strength of the DMI.** Colorized regions in phase diagram reproduced from Fig. 2a (main text) indicate phase boundary when $\mathbf{D}_{ij}/J$=0.1. Black squares and red circles indicate how these phase boundaries are shifted when $\mathbf{D}_{ij}/J$=0 and $\mathbf{D}_{ij}/J$=0.2, respectively.

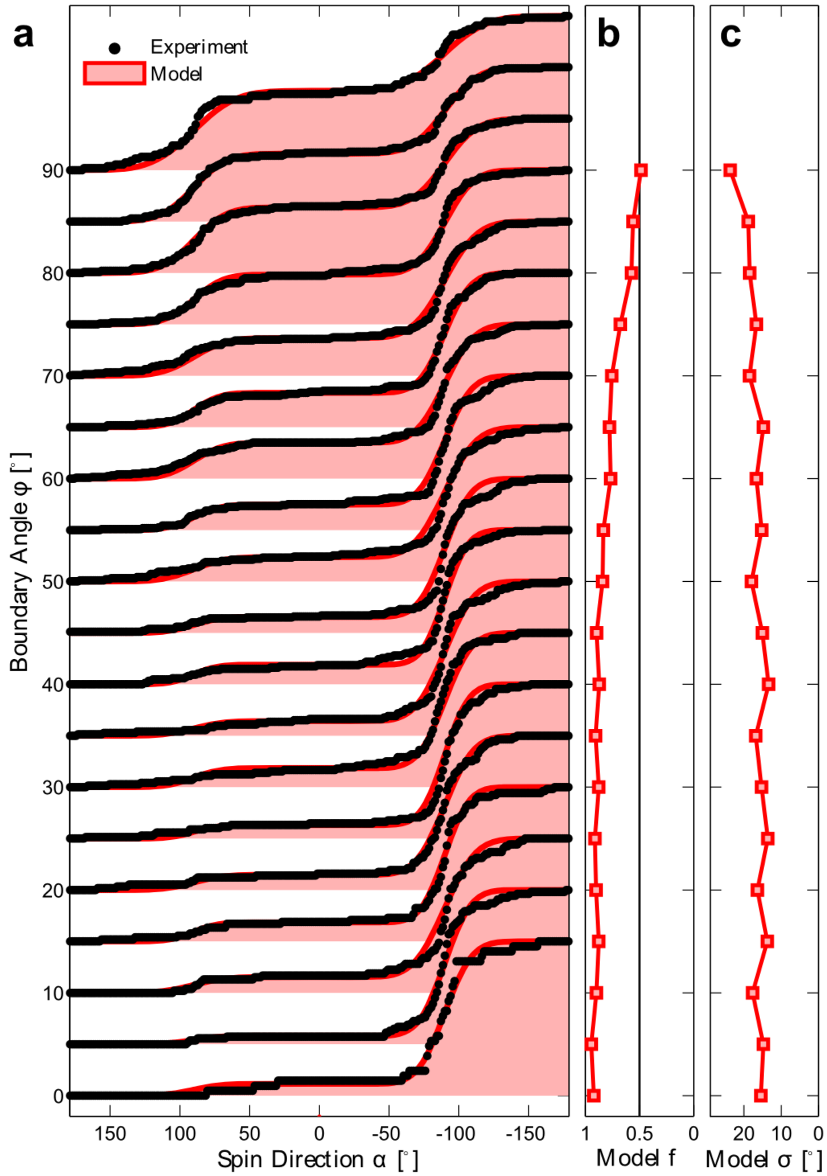


**Supplementary Figure 6 | Measuring spin texture chirality as a function of domain wall orientation $\phi$ with respect to substrate [001] direction.** (**a**) Integrated prevalence of domain wall magnetization $\alpha$ (along negative alpha direction) as a function of domain wall orientation $\phi$ (experimental data from two SPLEEM image sets, including the set of supplementary Fig. 2d and 4b). Steps at $\alpha = +90°$ and $\alpha = -90°$ indicate the contribution of counts from left-handedness and right-handedness,

respectively. The plots were fitted by a model where two normalized Gaussian peaks are considered for right-handed and left-handed chirality (cumulative sum of a Gaussian is the erf function). The probability of detecting domain walls with certain chirality can be written as $fe^{-(\alpha-90°)^2/2\sigma}/\sqrt{2\pi\sigma^2}+(1-f)e^{-(\alpha+90°)^2/2\sigma}/\sqrt{2\pi\sigma^2}$, where *f* is the fractional cumulative sum of the magnetic chirality histogram in the peak of $\alpha=+90°$ (right-handed chirality), relative to the total counts in both peaks. $f=0$ or $f=1$ means that all counts are in the first peak (left- handed chirality) or in the second peak (right-handed chirality), respectively. $f=0.5$ means that the counts at two peaks are same. The average magnetic chirality plotted in Fig. 4a in the main text is equal to 1-2*f*. *σ* is the width of the peaks in degrees, which corresponds to the standard deviation. We assume that both peaks have same width. (**b**) *ϕ*-dependent *f* indicates that domain walls gradually change from right-handed chiral wall at $\phi=0°$ to non-chiral wall at $\phi=90°$. (**c**) *ϕ*-dependent *σ* indicates that two peaks are about 15 to 20 degrees wide in all bins.

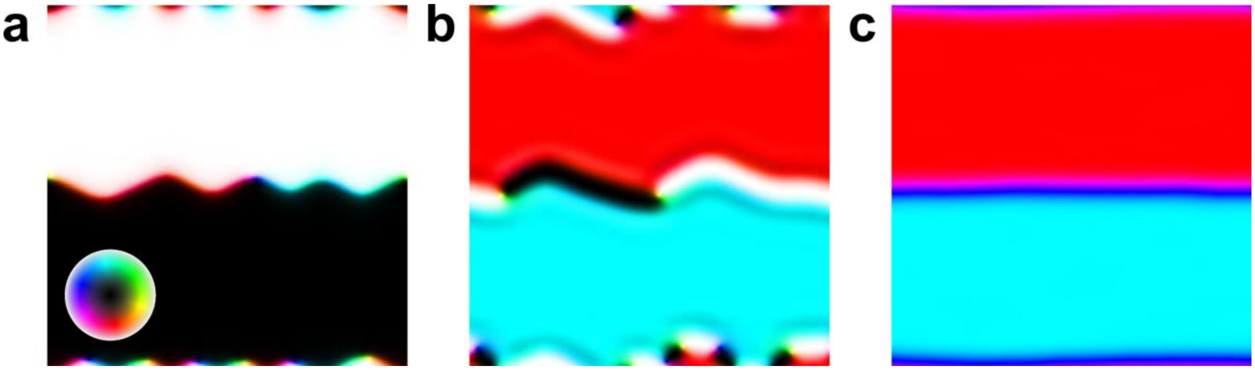


**Supplementary Figure 7 | Simulated spin configurations as a function of anisotropy, with vanishing DMI.** (**a**) $K_{eff}/J$=0.07 (**b**) $K_{eff}/J$=0.02 (**c**) $K_{eff}/J$=-0.11. $K_u/J$=0.05 is used for all three simulations. Colour wheel in **a** indicates the orientation of magnetization where black/white corresponds to $+z$/$-z$ direction. The magnetic chirality shown in Figs. 2b,e,c,f in the main text disappears in **a** and **b** due to the absence of the DMI. In contrast to the straight domain boundary shown in Figs. 2b,c in the main text, wavy boundary in **a** and **b** is induced by the interplay between dipolar energy and uniaxial anisotropy $K_u$: when the in-plane component of the magnetization within the domain wall is fixed by the easy axis of $K_u$, then the dipolar energy favours parallel domain configurations rather than head-to-head configurations.

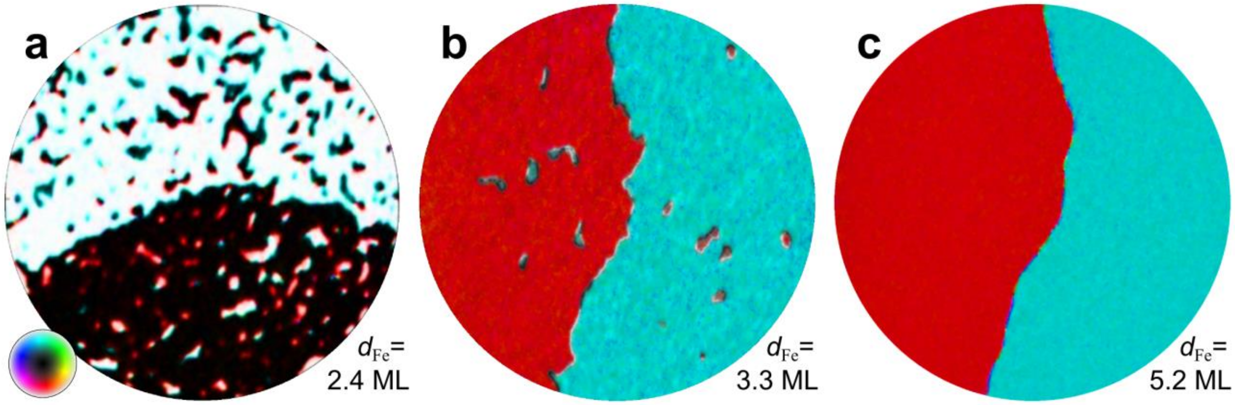


**Supplementary Figure 8 | Spin textures in Fe/15ML Ni/W(110) as a function of varying magnetic anisotropy by adjusting Fe thickness $d_{Fe}$.** Colour wheel (lower left of panel **a**) shows in-plane orientation of the magnetization, and black/white shows $+z/-z$ out-of-plane component. (**a**) At $d_{Fe}$=2.4ML cyan and red colour indicates right-handed Néel-type domain walls, along the W[001] direction, between black and white out-of-plane domains. (**b**) At $d_{Fe}$=3.3ML, black and white colour of the domain walls separating cyan and red domains indicates out-of-plane texture of the domain walls. (**c**) At $d_{Fe}$=5.2ML, green/yellow and purple colour of the domain walls indicates in-plane Néel type. Field of view of **a**-**c** is 10 μm.
